# Squaric Acid Bisphposphonates for Theranostics of Bone Metastasis – the Easy DOTA-Zoledronate

**DOI:** 10.3389/fnume.2022.870910

**Published:** 2022-05-10

**Authors:** Lukas Greifenstein, Nils Engelbogen, Domokos Máthé, Tilmann Grus, Frank Rösch, Ralf Bergmann

**Affiliations:** ^1^Curanosticum Wiesbaden-Frankfurt, Wiesbaden, Germany; ^2^Institute of Nuclear Chemistry, Johannes Gutenberg University, Mainz, Germany; ^3^Institute of Biophysics and Radiation Biology, Semmelweis University, Budapest, Hungary; ^4^Institute of Radiopharmaceutical Cancer Research, Helmholtz-Zentrum Dresden Rossendorf, Dresden Rossendorf, Germany

**Keywords:** bisphosphonates, theranostics, zoledronate, Ga-68 pet, metastasis

## Abstract

Bisphosponates are an interesting molecular class and in recent years their application has found its way into radiopharmaceutical research and thus into molecular imaging. In addition to great imaging of bone metastases, bisphospnate-based tracers for imaging also have some significant drawbacks. For example, their synthesis is often difficult. Additionally, this can lead to complex and almost impossible purification and quality control. This has limited the production and labeling of suitable molecular and their widespread use to a few facilities. Our squaric acid-based approach provides a way to overcome these problems and makes the synthesis as well as the purification of the compounds much easier. In addition, we were able to demonstrate that labeling with ^68^Ga is possible under the typical conditions.

## Introduction

Bone metastases are common in late stages of prostate, breast and lung cancer (1, 2). Up to 85% of the deceased patients who suffered from this sort of tumor diseases show this form of metastasis (3). Furthermore, it was shown that patients with bone metastases show increased mortality (1, 3). Bone metastases are formed when tumor cells of a primary tumor can penetrate the vascular system and enter the bone marrow from there. In the bone marrow, the tumor cells secrete growth factors that disturb the balance between osteoblasts and osteoclasts (4). In this context it is important to understand the structure of bone in general. It consists of a compact outer layer and a porous inner layer. The solid part of the bone consists of a network of collagen fibers surrounded by an inorganic mineral phase. This mineral phase, which makes up about 50–60% of the total bone, consists mainly of hydroxyapatite (HAP) (5). From a structural point of view HAP is a calcium-phosphate compound with the molecular formula Ca_10_(PO_4_)_5_(OH)_2_ (6).

Bisphosphonates (BP) are a stable modification of pyrophosphates and all have a P-C-P motif (7). In contrast to the P-O-P structure of pyrophosphates, this group has a high *in vivo* stability against enzymatic hydrolysis (8). All BP show an affinity toward HAP and therefore are only adsorbed in bone and not in any soft tissue (9). The affinity of bisphosphonates to HAP results from the complexation of Ca^2+^ in the HAP structure by the two phosphonate groups of the BP similar to the pyrophosphates. The insertion of an OH function as well as an amine function into the bisphosphonate structure can even increases the chemisorption of the compound to HAP, while nitrogen heterocycles increase bone resorption (8, 10). For use in radiopharmacy it is noteworthy that it is not the biological effect of BP that is decisive, but their tissue distribution and uptake into the target tissue, in this case bone metastases. Bisphosphonates bind preferentially to sites in bone with increased activity of bone remodeling (11, 12). Since bone metastases show increased activity of bone remodeling and bisphosphonates otherwise show little uptake into soft tissue and healthy bone, they are an excellent lead structure for the development of radiopharmaceuticals for the diagnosis and therapy of bone metastases.

In bisphosphonate-chelator conjugates the bisphosphonate serves as targeting vector and complexes different nuclides by means of a chelator and can thus be used for the diagnosis or therapy of bone metastases (13).

**Figure 1:**
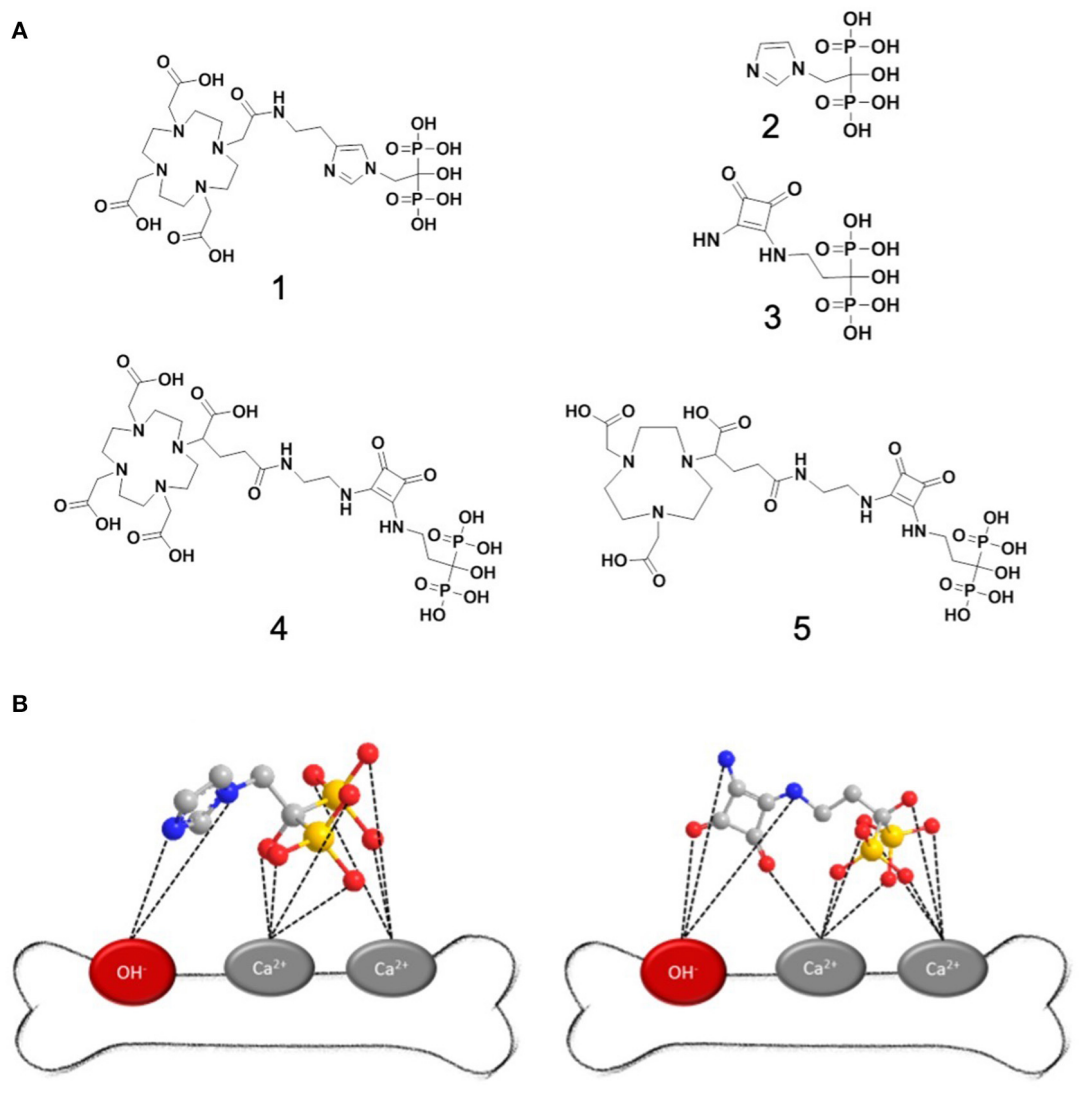
**(A)** Important structures of bisphosphonates for therapy and diagnosis of bone metastases: 1: DOTA-zoledronate 2: zoledronic acid 3: squaric acid pamidronate 4: DOTAGA.SA.PAM 5: NODAGA.SA.PAM; **(B)** left: binding hypothesis for zoledronic acid to hydroxyapatite; right: possible binding of the SA.PAM fragment. A comparable effect as for zoledronic acid is assumed.

For example, NOTA (1,4,7-triazacyclononane-1,4,7-triacetic acid) bisphosphonates are particularly suitable for diagnosis using PET, as they have better properties for complexing ^68^Ga than DOTA (1,4,7,10-tetraazacyclododecane-1,4,7,10-tetraacetic acid) derivatives (14) DOTA bisphosphonates, however, can complex a wider range of radiometals and are therefore suitable as theranostatic radiopharmaceuticals enabeling diagnosis and therapy with the same molecule (see Figure 1A). The latest development in the field of chelator-bisphosphonates is DOTA-ZOLEDRONATE (DOTA-ZOL, DOTA^ZOL^) (1) (15–18). It combines a chelator with a bisphosphonate of the latest generation and shows an increased bone uptake compared to chelator-bisphosphonate conjugates with simpler bisphosphonates such as BPAPD. However, problems are described for the synthesis of DOTA-ZOL: Unprotected bisphosphonates have a poor solubility in organic solvents and dissolve only in aqueous buffers. This makes it difficult to couple a chelator via an amide bond to an unprotected bisphosphonate, since most active esters tend to hydrolyze in aqueous buffers. Thus, for the synthesis of DOTA-ZOL, it is reported that coupling a DOTA-NHS ester with zoledronate leads to an increased amount of free DOTA as a hydrolysis product of the NHS ester. Consequently, separation of the free chelator becomes challenging (15). The solution would be synthesis of a protected BP which would require an increased synthetic effort.

Coupling by means of squaric acid esters offers a solution to these problems for several reasons: (i) Typically, for coupling with squaric acid esters, aqueous buffers can be used without any hydrolysis of the squaric acid ester. (ii) The squaric acid structure might play a similar role for binding to HAP as the imidazole in zoledronate (see Figure 1B) (3). (iii) The squaric acid structure is a good complexing agent and could form further bonds both to calcium and, via hydrogen bonds, to the OH groups in HAP. In this context several examples of beneficial influences of squaric acid coupling toward chemistry and biological behavior were reported recently (19–23).

For the detection of bone metastases in clinics, bone scan is still the most used method. The radionuclide Tc-99m used for this purpose is bound to BPs, such as hydroxymethylene bisphosphonate (HMDP), hydroxyethylene bisphosphonate (HEDP), methylene diphosphonate (MDP) or diphosphono-1,2-propanedicarboxylic acid (DPD), and the patient is examined using a gamma camera. General advantages of this method are good availability, moderate cost, and high sensitivity. Nonetheless, there are a variety of advantages using PET/CT scans over bone scans to image bone metastases. Especially the increased accuracy in detecting bone metastases, an additional increased sensitivity, better differentiation between benign and malignant tumors, higher specificity, multidimensional information, and higher resolution makes the more expensive PET/CT-scan a better tool for some investigations. In addition, the time spent by the patient is significantly reduced, on the one hand due to the reduced general examination time and on the other hand due to the potential need for further scans. As a result, patient management can be significantly improved.

## Materials and Methods

### Chemicals and Instrumentation

All chemicals were commercially available at Acros Organics (Nidderau, Germany), Merck (Darmstadt, Germany), Sigma Aldrich (Steinheim, Germany) or VWR (Darmstadt, Germany) and were used without further purification. Deuterated solvents for NMR spectroscopy were purchased from Deutero (Kastellaun, Germany). Silica gel (particle size: 0.040–0.063 mm) for column chromatography was purchased from VWR (Darmstadt, Germany). The measurements of ^1^H- and ^13^C-NMR spectra were performed on a Bruker Avance II 400 (400 MHz). Mass spectra were recorded on an Agilent Technologies 6130B Single Quadrupole LC/MS system. Semipreparative HPLC was performed on a Merck Hitachi LaChrom L-7100. Following column was used: Phenomenex Luna C18 (250 × 10 mm) 10 μm. Radioactivity was measured with a dose calibrator (ISOMED 2010; MED Nuklear-Medizintechnik Dresden GmbH, Dresden, Germany). Radio thin-layer chromatography (radio-TLC) was analyzed with a RITA^*^ TLC imager (Elysia-Raytest, Straubenhardt, Germany) and an evaluation software (GinaStar TLC; Elysia-Raytest, Straubenhardt, Germany).

### Radiolabeling

Labeling of compounds was performed in sodium acetate buffer (0.2 M, pH 4.5) with ^68^Ga eluate from a ^68^Ge/^68^Ga generator (iThembaLab, South Africa) followed by anionic postprocessing (0.6 M HCl). Labeling of NODAGA.SA.PAM and DOTAGA.SA.PAM was performed with 5, 10, and 15 nmol molecule in 450 μL of sodium acetate buffer (0.2 M, pH 4.5) with 50–100 MBq ^68^Ga in 150 μL eluate for 15 min at 95 °C. The pH of the labeling solution was adjusted to approximately 7, and the solution was used for injection after dilution with isotonic saline to the correct volume activity. Labeling of DOTA-ZOL was performed as described in previous literature (15–17). The analytical method for determining the radiochemical purity of the labeled chelator-bisphosphonate compounds was investigated by radio-TLC in a solution of acetone, acetylacetone and conc. hydrochloric acid (10:10:1). The labeled complexes remain at a Rf = 0, while unbound Ga-68 is detected as an acetylacetonate complex with a Rf = 1.0. Even if some methods for the determination of radiochemical purity by HPLC have been published, none of these methods could be used efficiently for the determination of radiochemical purity.

### *In vitro*-Stability Studies

Stability studies were performed in HS and PBS solution (pH adjusted to 7 by PBS buffer) in triplicate. HS (human male AB plasma, USA origin) were bought from Sigma Aldrich, phosphate buffered saline (PBS) pH = 7.4 was purchased from Sigma Aldrich as well. The final procedure used 50–70 μl of the labeling solution (5–10 MBq) added to 1 ml of either HS or PBS. The pH was controlled to ensure no influence of the labeling buffer on the solution. Radio-TLC in a solution of acetone, acetylacetone and conc. hydrochloric acid (10:10:1) was used to determine the stability.

### *In vivo*-Experiments

The Wistar rats were housed in the animal facility of the Helmholtz-Zentrum Dresden-Rossendorf and experiments were performed according to the guidelines of the European and German Regulations for Animal Welfare approved by the local Ethics Committee for Animal Experiments (Landesdirektion Dresden; file numbers 24-9165.40-4/2013, 24-9168.21-4/2004 1).

Male Wistar rats (RjHan:WI, Janvier Labs, France), 5–7 weeks old, were kept in a pathogen-free facility with ad libitum access to water and food. Biodistribution studies were performed with [^68^Ga]Ga-NODAGA.SA.PAM (*n* = 4, 5.8 MBq/animal) and [^68^Ga]Ga-DOTA-ZOL (*n* = 4, 6.9 MBq/animal). Animals were sacrificed at 5 min and 60 min post-injection (p.i.). Blood and the major organs were collected, weighed, and counted in a cross-calibrated γ-counter (Isomed 1000, Isomed GmbH) and Wallac WIZARD Automatic Gamma Counter (PerkinElmer). The activity of the tissue samples was decay-corrected and calibrated by comparing the counts in tissue with the counts in aliquots of the injected radiotracer that had been measured in the γ-counter at the same time. The activity in selected organs, that could be completely extracted, was expressed as percent-injected activity per organ (%ID) and the activity concentration in tissues and organs as standardized uptake value (SUV in [MBq activity/g tissue] / [MBq injected activity/g body weight]). Values are quoted as mean ± standard deviation for each group of animals. PET scans were performed using dedicated rodent PET/CT scanner (NanoPET/CT, Mediso, Hungary). The PET experiments were carried out with rats under general anesthesia that was induced and maintained by inhalation of 12% and 9% (v/v) desflurane in 30/10% (v/v) oxygen/air, respectively. Anesthetized rats were positioned on a warmed bed along the scanner axis. The ^68^Ga-labeled radiotracers in 300 μL isotonic NaCl were infused over 1 min into a tail vein. PET images were acquired beginning with the injection over 2 h and were reconstructed in dynamic mode with 36 frames and 0.5 mm^3^ voxel size. Region-of-interest (ROI) quantification was performed with ROVER (ABX GmbH, Germany). The ROI values were corrected for recovery and partial volume effects. The values were expressed as SUV_mean_.

### Syntheses

#### DOTAGA.SA

NH_2_-DOTAGA (30 mg; 58 μmol; 1 eq.) and squaric acid diethyl ester (26 μL; 30 mg 176 μmol; 3 eq.) were dissolved in phosphate buffer (0.5 M; pH 7; 0.5 mL) and stirred for 2 h at RT. The pH value of the reaction was controlled and, if necessary, adjusted to pH 7–7.5 with sodium hydroxide solution (1 M). The product DOTAGA.SA (28 mg; 43.5 μmol; 75 %) was isolated by semi-preparative HPLC (column: Phenomenex Luna C18 (250 x 10 mm) 10 μm; flow rate: 5 mL/min; solvent: H_2_O/MeCN +0.1 % TFA; gradient: 0–18 % MeCN in 15 min; R_t_ = 13 min) and obtained as a colorless solid after lyophilisation. ^1^H-NMR (600 MHz, D_2_O): δ (ppm) = 1.30 (dt: 3H, J3HH = 7.1 Hz; J = 12 Hz; CH_3_); 1.81 (br: 2H; CH2); 2.37 (br: 2H; CH_2_); 2.77–3.99 (m: 27H); 4.58 (dq: 2H; J3HH = 7.1 Hz; J = 21 Hz; CH_2_-CH_3_). ^13^C-NMR (151 MHz, D_2_O): δ (ppm) = 15.01 (CH_3_); 33.04 (CH_2_-CH2); 39.45 (CH_2_-CH_2_); 39.45 (CH_2_-CH_2_); 39.75 (CH_2_-CH_2_); 43.62 (CH_2_-CH_2_); 43.80 (CH_2_-CH_2_); 55.05 (CH_2_); (70.64 (CH_2_-CH_3_); 113.32 (COOH); 115.26 (COOH); 117.19 (COOH); 119.12 (COOH); 162.54 (CH_2_); 162.78 (CH_2_); 163.25 (CH_2_); 173.74 (CO-NH); 176.75 (C.SA); 177.22 (C.SA); 183.34 (C.SA); 188.72 (C.SA). MS (ESI positive): m/z (%): 322.2 [M+2H]^2+^; 643.3 [M+H]^+^; [M] calculated: 642.29; UPLC (Gradient: 0-100 % B in 15 min): [M] R_t_ = 3.01 min.

#### NODAGA.SA

NH_2_-NODAGA (5 mg; 12 μmol; 1 eq.) and squaric acid diethyl ester (9 μL; 10 mg 60 μmol; 5 eq.) were dissolved in phosphate buffer (0.5 M; pH 7; 0.5 mL) and stirred for 2 h at RT. The pH value of the reaction was controlled and, if necessary, adjusted to pH 7–7.5 with sodium hydroxide solution (1 M). The product NODAGA.SA (6 mg; 11 μmol; 93 %) was isolated by semi-preparative HPLC (column: Phenomenex Luna C18 (250 × 10 mm) 10 μm; flow rate: 5 mL/min; solvent: H_2_O/MeCN + 0.1 % TFA; gradient: 0–30 % MeCN in 20 min; Rt = 13.1 min) and obtained as a colorless solid after lyophilisation.^1^H-NMR (300 MHz, D_2_O): δ (ppm) = 1.32 (dt: 3H, J3HH = 7 Hz; J = 7 Hz; CH_3_), 1.77–2.02 (m: 2H; CH_2_); 2.29 (dt; 2H; J3HH = 7.5 Hz; J3HH = 6.6 Hz; CH-CH_2_-CH_2_); 2.85-3.01 (m: 4H; cyclic-CH_2_); 3.02–3.22 (m: 8H; cyclic-CH_2_); 3.25–3.35 (m: 2H; CH_2_); 3.40–3.53 (m: 2H; CH_2_); 3.57–3.63 (m: 1H; CH); 3.78 (br: 4H; CH_2_-COOH); 4.61 (dq: 2H; J3HH = 7.1 Hz; J = 13 Hz; CH_2_-CH_3_). MS (ESI positive): m/z (%): 542.2 [M+H]^+^; 1084.3 [2M+H]^+^; [M] calculated: 541,24; UPLC (Gradient: 0–100 % B in 15 min): [M] Rt = 3.38 min.

#### Pamidronate

β-Alanine (2.2 g, 25 mmol, 1 eq.) and phosphonic acid (4.1 g; 50 mmol; 2 eq.) were dissolved in sulfolane and then phosphorus trichloride (4.4 mL; 6.9 g; 50 mmol; 2 eq.) was added dropwise within 15 min. The reaction mixture was then stirred for 3 h at 75°C. The mixture was cooled to 0°C and diluted with water (25 mL). The mixture was then stirred for another 12 h at 105°C. Via crystallization by addition of ethanol (20 mL) and cooling of the reaction mixture to 0°C, pamidronate (2.1 g; 8.9 mmol; 36%) was obtained.^1^H NMR (300 MHz, D_2_O): δ (ppm) = 2.20 (tt: J3HH = 6.2 Hz; J3PH = 13 Hz; 2H; NH_2_-CH_2_-CH_2_); 3.27 (t: J3HH = 6.2 Hz; 2H; NH_2_-CH_2_). ^31^P NMR (121.5 MHz, D_2_O): δ (ppm) = 17.5 (s, 2P). MS (ESI positive): m/z (%): 236.0 (100) [M+H]^+^; 471.0 (25) [2M+H]^+^; (ESI negative): 233.9 (100) [M-H]^−^; 469.0 (90) [2M-H]^−^; 703.9 (80) [3M-H]^−^; 939.0 (70) [4M-H]^−^; 1174.0 (30) [5M-H]^−^; 1409.0 (5) [6M-H]^−^; [M] calculated: 235.0. UPLC (gradient: 0-30% B in 4 min): [M] Rt = 0.32 min.

#### NODAGA.SA.PAM

NODAGA.SA (19.6 mg; 36.2 μmol; 1 eq.) was dissolved with pamidronate (10 mg; 42.55 μmol; 1.2 eq.) in phosphate buffer (0.5 M; pH 9; 1 mL) and stirred for 24 h at RT. The pH of the reaction was monitored and adjusted to pH 9–10 with sodium hydroxide solution (1 M) if necessary. By semipreparative HPLC (column: Phenomenex Luna C18 (250 × 10 mm) 10 μm; flow rate: 5 mL/min; running medium: H_2_O/MeCN + 0.1% TFA; gradient: 5–6.5% MeCN in 9 min; Rt = 7.5 min), the product NODAGA.SA.PAM (8 mg; 10.96 μmol; 30%) was isolated and obtained as a colorless solid after lyophilization.^1^H NMR (300 MHz, D_2_O): δ (ppm) = 1.87–2.03 (m: 2H; CH_2_); 2.09-2.38 (m: 2H; CH_2_); 2.85-3.04 (m: 4H; ring-CH_2_); 3.05–3.25 (m: 8H; ring-CH_2_); 3.27-3.39 (m: 2H; CH_2_); 3.49 (t: 1H; J3HH = 7 Hz; CH); 3.57–3.70 (br: 2H; CH_2_); 3.78 (br: 4H; CH_2_-COOH); 4.61 (dq: 2H; J3HH = 7.1 Hz; J = 13 Hz; CH_2_-CH_3_). ^31^P NMR (121.5 MHz, D_2_O): δ (ppm) = 18.20 (s, 2P). MS (ESI positive): m/z (%): 366.2 (35) [M+2H]^2+^; 731.1 (100) [M+H]^+^; [M] calculated: 730.2; [M(^nat^Ga)]: 399.2 (80)/400.1 (75) [M(Ga)+2H]^2+^; 797.0 (100)/799.0 (60) [M(Ga)+H]^+^; calculated: 796.2 (100)/798.2 (65). UPLC (gradient: 0–100% B in 15 min): [M] Rt = 0.81 min; [M(Ga)] Rt = 0.89 min.

#### DOTAGA.SA.PAM

DOTAGA.SA (27 mg; 42 μmol; 1 eq.) was dissolved with pamidronate (24.7 mg; 105 μmol; 2.5 eq.) in phosphate buffer (0.5 M; pH 9; 1 mL) and stirred for 24 h at RT. The pH of the reaction was monitored and adjusted to pH 9 to 10 with sodium hydroxide solution (1 M) if necessary. By semipreparative HPLC (column: Phenomenex Luna C18 (250 × 10 mm) 10 μm; flow rate: 5 mL/min; running medium: H_2_O/MeCN + 0.2% TFA; gradient: 0–8% MeCN in 12 min; Rt = 11 min), the product DOTAGA.SA.PAM (17 mg; 20.5 μmol; 49%) was isolated and obtained as a colorless solid after lyophilization. ^1^H NMR (300 MHz, D_2_O): δ (ppm) = 1.88 (br: 2H; CH_2_); 2.12–2.36 (m: 2H; CH_2_); 2.47 (br: 2H; CH_2_); 2.90–4.00 (m: 29H). ^31^P NMR (121.5 MHz, D_2_O): δ (ppm) = 18.51 (s, 2P). MS (ESI positive): m/z (%): 416.7 (50) [M+2H]^2+^; 832.2 (100) [M+H]^+^; [M] calculated: 831.25; [M(^nat^Ga)]: 449.7 (100)/ 450.5 (65) [M(Ga)+2H]^2+^; 898.2/899.2 (5) [M(Ga)+H]^+^; calculated: 897.25 (100)/899.25 (65); [M(^nat^Lu)]: 335.5 (20)/ 450.5 (65) [M(Lu)+3H]^3+^; 502.6 (100) [M(Lu)+2H]2+; calculated: 1003.25; UPLC (gradient: 0-10% B in 4 min): [M] Rt = 0.48 min; [M(Ga)] R_t_ = 0.55 min; [M(Lu)] Rt = 0.56 min.

## Results and Discussion

### Synthesis, Labeling and Stability

**Figure 2:**
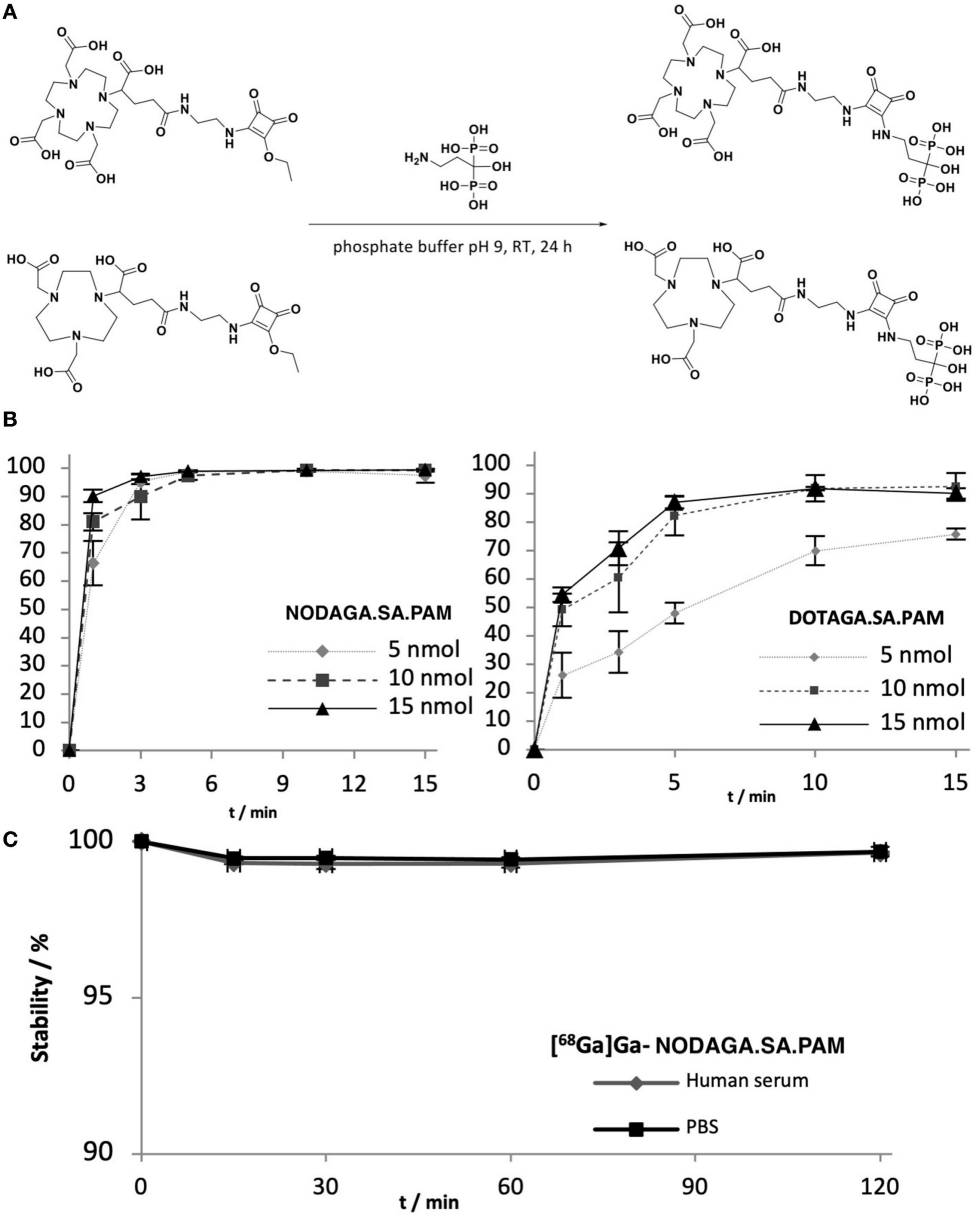
**(A)** Synthesis scheme for the reaction between DOTAGA.SA/NODAGA.SA with pamidronate. Both reactions take place under the same conditions (phosphate buffer (0.5 M, pH 9), rt, 24 h). **(B)** left: labeling kinetics of DOTAGA.SA.PAM with ^68^Ga with different amounts of precursor. right: labeling kinetics of NODAGA.SA.PAM with ^68^Ga **(C)** *in vitro* stability of [^68^Ga]Ga-NODAGA.SA.PAM in human serum and PBS buffer.

The synthesis of DOTAGA.SA.PAM (4) and NODAGA.SA.PAM (5) are straight forward and comparable to the procedures already published (19–23). First the chelate.SA motif is prepared and afterwards the bisphosphonate pamidronate (PAM) is coupled at a pH of 9 in phosphate buffer (see Figure 2A). Typically, the hydrolysis of chelator-active esters during coupling to amine bisphosphonates is a known problem and complicates the purification of the desired compound (15). However, this is not a problem when coupling to SA-derivatives as they can be purified with a straightforward preparative HPLC set up described in the experimental section. Labeling kinetics were performed for the two compounds NODAGA.SA.PAM and DOTAGA.SA.PAM under typical conditions. For labeling n.c.a. ^68^Ga was eluted with 0.6 M HCl from an iThembaLab ^68^Ge/^68^Ga generator and purified via the corresponding cationic acetone post-processing (9, 24). Different amounts of each precursor (5, 10, 15 nmol) were dissolved in 450 μL of sodium acetate buffer (0.2 M, pH 4.5) and heated to 95°C. After addition of 100 – 120 μL of N2 solution from the post-processing containing approximately 50 MBq of ^68^Ga the reaction started. At fixed time points (1, 3, 5, 10, and 15 min), aliquots of the reaction solution were taken. The analytical method for determining the radiochemical purity of the ^68^Ga labeld chelator-bisphosphonate compounds was investigated by radio-TLC in a solution of acetone, acetylacetone and conc. hydrochloric acid (10:10:1). The labeled complexes remain at a R_f_ value of 0.0, while uncomplexed ^68^Ga is found as an acetylacetonate complex with a R_f_ value of 1.0. Since the complexes of the bisphosphonate compounds show insufficient retention on the available HPLC columns, no further analysis by radio-HPLC was carried out. The NOTA-based bisphosphonate NODAGA.SA.PAM shows better radiochemical yields and faster kinetics for ^68^Ga labeling than the DOTA-based DOTAGA.SA.PAM (see Figure 2B). This result is in line with expectations, as NODAGA is better suited for the complexation of Ga^3+^ than DOTAGA (25–27). For NODAGA.SA.PAM, quantitative radiochemical yields can be achieved in a short time even with small amounts of substance. With DOTAGA.SA.PAM radiochemical yields of up to 90% can be obtained. Both compounds were investigated *in vitro* in human serum and PBS buffer and provided excellent stabilities with values >95% over 2 h. However, it is noteworthy that this stability only gives insights into desorption of ^68^Ga and does not evaluate the metabolism of the probe.

### *In vivo* Experiments

**Figure 3:**
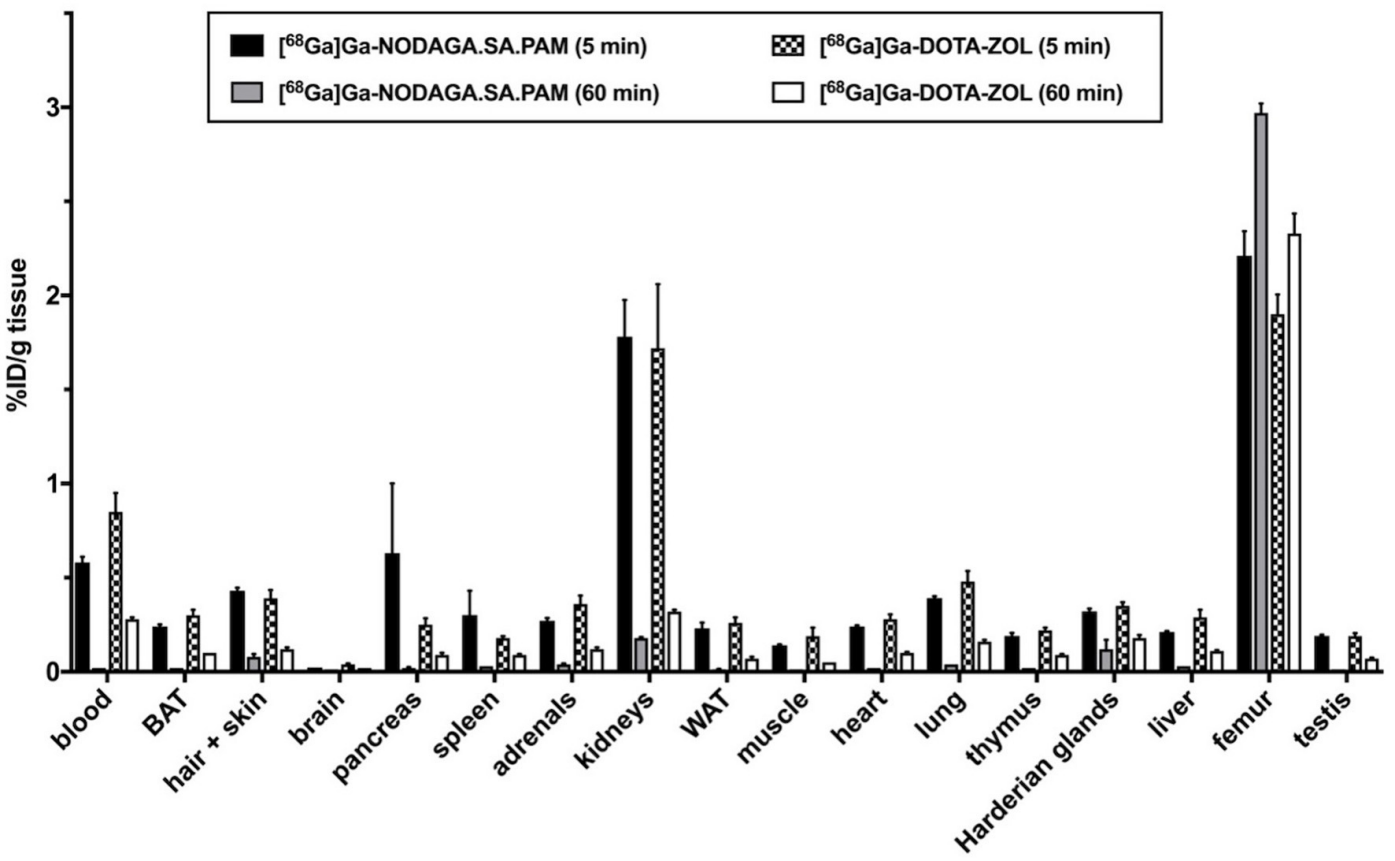
*Ex vivo* biodistribution of the [^68^Ga]Ga-NODAGA.SA.PAM and [^68^Ga]Ga-DOTA-ZOL expressed as activity concentration (%ID/g tissue, mean ± SEM, *n* = 4) at 5 and 60 min after single intravenous injections in Wistar rats. BAT, brown adipose tissue; WAT, white adipose tissue.

**Figure 4:**
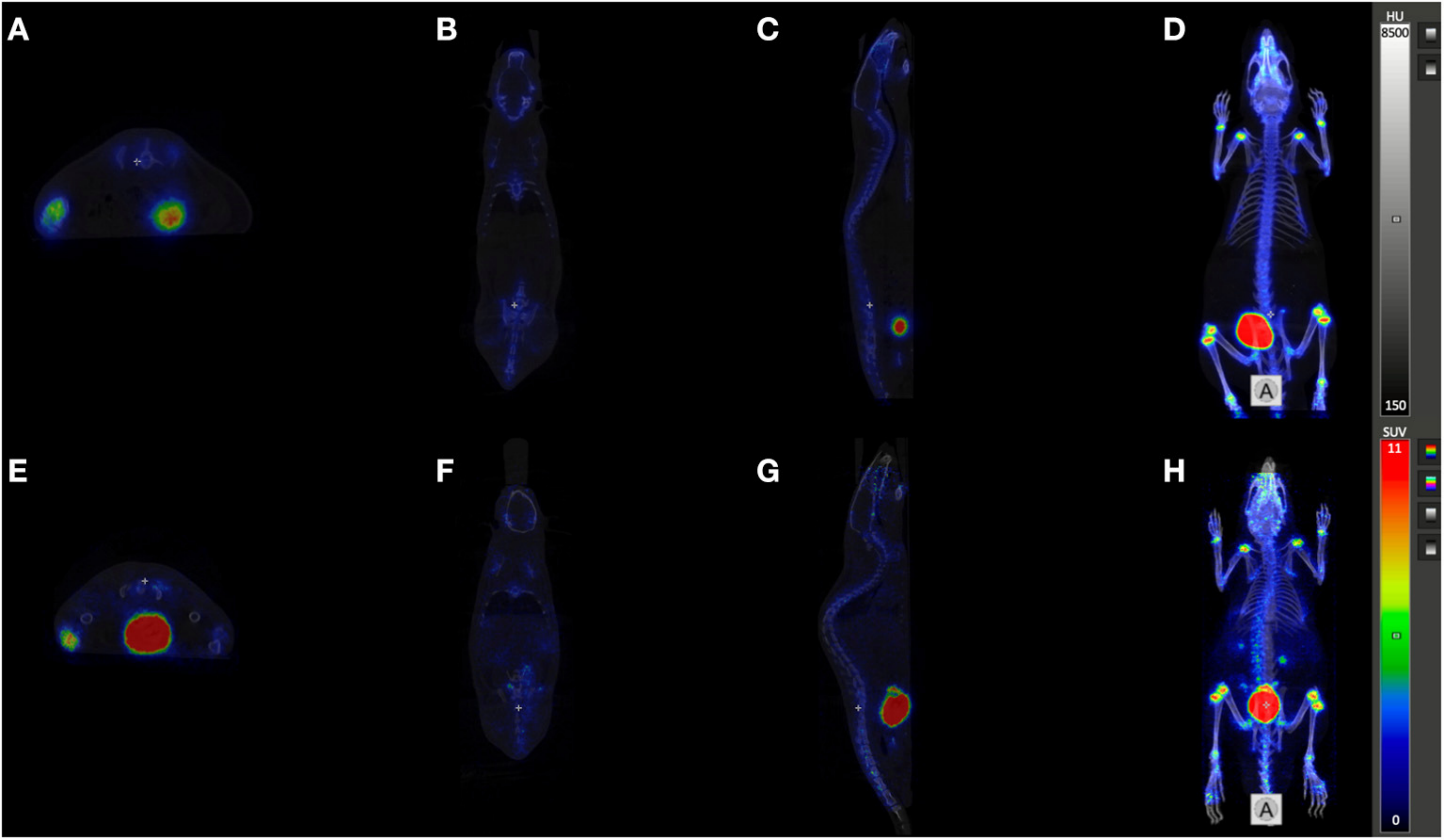
PET/CT imaging: Orthogonal sections **(A)**, E transaxial, **(B)**, F coronal, **(C)**, G sagittal, and Maximum Intensity Projections **(D, H)** of registered PET/CT studies (midframe time 90 min p.i.) of [^68^Ga]Ga-NODAGA.SA.PAM **(A**–**D)** and [^68^Ga]Ga-DOTA-ZOL **(E–H)**.

For NODAGA.SA.PAM, further *in vivo* studies were performed in young, healthy wistar rats. For comparisons to an established bisphosphonate compound, DOTA-ZOL was investigated under the same conditions. For both compounds, biodistributions were established after 5 and 60 min p.i. (see Figure 3). In addition, a dynamic PET scan was recorded for each compound over 120 min (see Figure 4). Biodistribution shows that both derivatives have a high uptake and retention in bone, with a slightly higher uptake of [^68^Ga]Ga-NODAGA.SA.PAM (2.97 ± 0.10 %ID/g) in comparison to [^68^Ga]Ga-DOTA-ZOL (2.33 ± 0.21 %ID/g). In addition, both compounds show renal excretion and only little retention in the remaining tissue. Furthermore, it is noteworthy that after 5 min there is significant higher uptake for NODAGA.SA.PAM into pancreas and spleen compared DOTA-ZOL. This uptake decreases after 60 min which suggests that it is not a specific accumulation. Particularly important besides the general uptake into the bone is the specific uptake into a bone area with increased bone remodeling rate. This area is represented by the epiphyses in young, still growing rats.

The PET images (midframe time 90 min) show that both compounds have increased retention in the epiphyses, again confirming specific uptake into areas of active bone remodeling. Apart from the skeleton only the bladder is otherwise highlighted. Thus, there is no retention of the bisphosphonates in the soft tissue and both compounds have a preferential renal excretion. [^68^Ga]Ga-NODAGA.SA.PAM (40 ± 4 %ID_renal_, 60 min p.i.) has a slightly higher renal excretion compared to [^68^Ga]Ga-DOTA-ZOL (33 ± 17%ID_renal_, 60 min p.i.) (see Figure 3). A closer look at the uptake into the epiphyses shows that [^68^Ga]Ga-NODAGA.SA.PAM (SUV_Epiphyses_ = 22.9) has a higher accumulation than [^68^Ga]Ga-DOTA-ZOL (SUV_Epiphyses_ = 17.4). This and the improved renal excretion result in a higher epiphysis-to-blood ratio ([^68^Ga]Ga-DOTA-ZOL = 30.3; [^68^Ga]Ga-NODAGA.SA.PAM = 299.1) and thus a better image contrast. Similar results of an improved bone-to background ratio were obtained by Pfannkuchen et al. for [^68^Ga]Ga-NODAGA-ZOL compared to [^68^Ga]Ga-DOTA-ZOL (28). The ^68^Ga complexes of the NODAGA-based compounds show a significantly higher femur-to-blood and femur-to-muscle ratio than the DOTA-based DOTA-ZOL, with NODAGA.SA.PAM achieving the best contrast. This finding of a low femur uptake for DOTA-ZOL is also well in line with literature (15, 29), however there are also results with other bone seeking agents like DOTA-Bn-SCN-HBP or Ga-DOTA-(D-Asp)^n^ reporting higher femur accumulation (30, 31). In conclusion, we present NODAGA.SA.PAM, a molecule that, in addition to its facilitated synthesis compared to the established DOTA-ZOL, also shows improved *in vivo* behavior. In biodistribution studies as well as in the *in vivo* PET images an increased uptake into bone and especially into the epiphyses was demonstrated. Furthermore, the higher epiphyses -to-blood ratio resulted in better imaging quality. In addition, we also introduced DOTAGA.SA.PAM, a promising molecule with a simple synthetic strategy that can be used for labeling with both ^68^Ga and ^177^Lu. With this molecule, we hope to take a further step toward a simple diagnosis and therapy of bone metastases in the future. However, labeling and *in vivo* potential remains to be demonstrated.

## Data Availability Statement

The raw data supporting the conclusions of this article will be made available by the authors, without undue reservation.

## Ethics Statement

The animal study was reviewed and approved by Landesdirektion Dresden; file numbers 24-9165.40-4/2013, 24-9168.21-4/2004 1.

## Author Contributions

LG, NE, and RB: conceptualization. LG, NE, DM, and RB: methodology and formal analysis. LG, NE, TG, DM, and RB: software. NE and LG: investigation. LG, RB, and DM: data curation. LG and TG: writing—original draft preparation. LG, NE, DM, TG, FR, and RB: supervision. FR and RB: writing—review and editing. All authors contributed to the article and approved the submitted version.

## Conflict of Interest

The authors declare that the research was conducted in the absence of any commercial or financial relationships that could be construed as a potential conflict of interest.

## Publisher's Note

All claims expressed in this article are solely those of the authors and do not necessarily represent those of their affiliated organizations, or those of the publisher, the editors and the reviewers. Any product that may be evaluated in this article, or claim that may be made by its manufacturer, is not guaranteed or endorsed by the publisher.
